# GPS navigation assistance is associated with driving mobility in older drivers

**DOI:** 10.1371/journal.pdig.0000768

**Published:** 2025-04-03

**Authors:** Sol Morrissey, Stephen Jeffs, Rachel Gillings, Mizanur Khondoker, Anuraj Varshney, Mary Fisher-Morris, Ed Manley, Michael Hornberger

**Affiliations:** 1 Norwich Medical School, University of East Anglia, Norwich, United Kingdom; 2 Department of Psychology, University of Exeter, Exeter, United Kingdom; 3 Oxford Brookes University, Oxford, United Kingdom; 4 MemCheck Memory Clinic, Chester Wellness Centre, Chester, United Kingdom; 5 School of Geography, University of Leeds, Leeds, United Kingdom; Yonsei University College of Medicine, KOREA, REPUBLIC OF

## Abstract

Maintaining driving mobility is essential for maintaining independence and wellbeing within older age. However, cognitive decline caused by age-related neurophysiological changes typically causes older drivers to self-regulate their driving and reduce their driving mobility. Electronic navigation assistance technologies, such as Sat-Nav, are increasingly popular amongst older drivers and can potentially alleviate cognitive demands amongst older drivers to enhance driving mobility. Yet despite the growing usage of navigation assistance technologies amongst older drivers, little research has been conducted to establish how and when they are used by older drivers, and it is not known whether they can offset cognitive decline to promote driving mobility. 895 older drivers (mean age: 71.04) were recruited for a prospective cohort study. Participants self-reported their navigation assistance usage as well as their driving mobility (frequency, space), before completing objective cognitive assessments (allocentric and egocentric orientation, recognition and source memory) and a subjective spatial orientation ability questionnaire. We establish profiles of older driver navigation assistance usage, showing that a considerable majority of older drivers use navigation assistance - with the majority using it for some journeys, and most commonly for the entire journey to a new destination. We show that navigation assistance usage is associated with worse subjective orientation ability, but not objective cognitive performance, and is positively associated with greater driving mobility. Importantly, we demonstrate that within individuals with poor wayfinding ability, navigation assistance usage is associated with increased driving mobility. In conclusion, navigation assistance usage is associated with increased driving mobility within healthy older drivers and is relied upon more by individuals with lower wayfinding confidence. As navigation assistance devices can specifically enhance driving frequency in individuals with worse wayfinding ability, who are more likely to reduce their driving, they should be recommended to older adults and integrated into comprehensive strategies for promoting driving independence in the older adult population.

## Author summary

Maintaining driving mobility in older age is important for quality of life and wellbeing. However, as people age, worsening spatial abilities typically lead to reduced driving. GPS devices can potentially help mitigate navigation concerns, as they provide turn-by-turn navigation assistance while driving. However, it is not currently well understood how commonly they’re used within older adult populations, whether they impact driving mobility (i.e., how frequently and far someone drives), or whether GPS usage relates to either subjective or objective cognitive performance in older age. Here, we investigated how GPS usage related with driving mobility and cognitive performance within 895 older drivers. We found that older adults with a poorer sense of direction rely more on GPS navigation assistance. Importantly, those who use GPS tend to drive more frequently than those who do not, suggesting that these tools help mitigate against spatial orientation difficulties and help maintain driving mobility. As our study shows that using GPS navigation tools is associated with better driving mobility, supporting older adults in using GPS devices may help older drivers with a reduced sense of direction maintain their driving.

## Introduction

Driving is the preferred method of transportation amongst older adults, and is vital for maintaining independence as well as physical, social, and cognitive health in older age [1,2]. Nonetheless, due to neurophysiological changes that take place in healthy ageing, many older adults typically self-regulate their driving behaviour by reducing their driving mobility, such as the frequency and distance of travel [3]. Due to the ageing population, there is an increased urgency to understand factors that may be influencing driving mobility within older adult populations to ensure that older adults can meet their mobility requirements and drive safely for longer.

Electronic navigation assistance systems, such as satellite navigation, integrate global positioning system (GPS) vehicle location information with digital maps to provide drivers with a sequence of steps that enable the driver to navigate an optimal route to their chosen destination. Among older age drivers, GPS use is rising in alignment with the increased prevalence of smartphones amongst older adult populations [4]. Operating a vehicle necessitates the intricate coordination of cognitive and physical abilities, and GPS can alleviate the cognitive demands of navigation, thereby enhancing driving performance [5]. This has led to the proposal to optimise in-vehicular technologies, such as GPS, to potentially offset age-related impairments in older adults to improve their driving safety and enable for greater mobility [6–8].

Research on GPS use in older adult populations to date has focussed upon attitudes and safety concerns, with GPS usage generally being positively associated with high usability and improved perceptions of safety [8–14]. Within older adults, GPS usage has been found to increase willingness to travel to unfamiliar areas. Compared to younger adults, older adults use GPS more frequently and input a greater number of destinations [6,15]. However, it remains unclear among older adult populations how prevalent GPS usage is, which driving situations are associated with GPS usage, whether GPS usage influences driving mobility, and how the prevalent age-related cognitive changes to memory and spatial navigation might influence GPS usage.

During the ageing process, changes to both memory performance and spatial navigation ability result in increased difficulty during wayfinding [16]. Indeed, navigation concerns have been prominently reported in older age drivers, particularly in unfamiliar areas, and they are more likely to show worse performance in wayfinding activities compared to younger drivers [17,18]. Furthermore, individuals with a poor sense of direction are more likely to reduce their driving [12,19,20], and recent research from our group has shown that spatial navigation performance is the main cognitive component associated with driving frequency and difficulty in older age [21].

It is also not yet understood how GPS usage in older drivers is related to cognitive performance, and in which situations older adults typically use GPS. To date, research assessing the effects of GPS on cognition has largely focused on younger populations and non-driving GPS use.

The current study addresses these research shortcomings by establishing the driving mobility patterns of GPS usage in a large sample of community-dwelling older adult drivers, and how this relates to their cognitive changes. Finally, we will explore whether GPS allows to ameliorate cognitive changes in driving mobility of older drivers. Our specific aims are to establish i) the prevalence and demographic profiles of older drivers who use GPS across different driving situations and how this relates to other in-vehicle technologies; ii) whether GPS usage is associated with changes to driving frequency and space; iii) how GPS usage is related to objective and subjective cognitive measures of spatial navigation and episodic memory, and iv) whether GPS usage enables individuals with worse wayfinding ability to have a greater driving mobility. We hypothesise that i) drivers further along the older age spectrum will be most likely to use GPS and other in-vehicle technologies more frequently; ii) older drivers who use GPS will drive more frequently and at a greater driving space; iii) older drivers with worse spatial navigation and memory performance will use GPS more frequently and in more familiar environments; and iv) individuals with worse wayfinding performance who use GPS will have greater driving mobility than those who do not use GPS.

## Materials and methods

### Participant recruitment

895 older adults (mean age: 71.04, 514 female) were recruited between February 2021 and August 2021 to complete the study. The inclusion criteria for the study were being age 65 or older, having a current driving license, and being a regular driver (driving once per week minimum). The exclusion criteria for the study were not driving regularly, having a medical condition that contraindicates driving, having an untreated significant visual or physical impairment, having a diagnosis of mild cognitive impairment or dementia, taking medications for dementia, and high alcohol consumption (> 45 units per week). Participants were recruited via online and media advertisement. Signed informed consent was obtained from each participant prior to conducting the experimental protocol and data was attributed anonymously. Ethical approval for the study was provided by the Faculty of Medicine and Health Sciences Research Ethics Committee at the University of East Anglia (FMH2019/20-134).

### Procedure

Participants initially completed questionnaires online related to their demographic information (age, gender, education), health, driving history, driving habits, and a custom questionnaire on navigation ability. Following this, participants completed an online neuropsychological testing battery assessing cognitive performance. The cognitive tests used within this study included the Virtual Supermarket Task, measuring for allocentric and egocentric orientation performance, and a Picture Recognition task, measuring recognition memory and source memory. For detailed information on all cognitive tasks within the testing battery, see [22].

### GPS frequency, situational usage, and wider technology usage

Within the driving history questionnaire, participants were asked “Which of the following in-car technology do you use?” If participants selected Sat-Nav (dedicated device) or Sat-Nav (app on mobile phone), they were asked “How often do you use (in-car technology)?” (No use – Rarely – Some Journeys – Most Journeys – Every time I drive). This comprised the GPS frequency measure. The number of non-navigation assistance technological items participants used (i.e., Bluetooth audio device, Cruise control, Lane control, Parking assistance, or Other) were totalled for the technology usage measure. Participants were also asked, “In which of the following situations do you use Sat-Nav?” (I do not use Sat Nav – If I get lost on a new route – As a backup in case I forget a planned route – When following a new route to a familiar destination – The entire journey, when driving to a new destination – The entire journey, when driving along a familiar route). The most GPS dependent situation was coded for each participant (“I do not use Sat Nav” = least dependent, “The entire journey, when driving along a familiar route” = most dependent). This comprised the GPS situational usage measure.

### Driving, Orientating, and Navigating questionnaire (DON)

We developed the DON, a custom driving-based navigation questionnaire, to measure an individual’s subjective orientation performance and spatial strategy when driving and navigating (see S1 Appendix). Previous work has shown that the DON correlates with the Santa Barbara Sense of Direction of Scale (SBSOD) [22]. The DON comprised of five items related to landmark-based navigation strategies, seven items related to allocentric navigation strategies, and four items related to egocentric navigation strategies. These items were totalled for each category. The total DON score comprised a subjective orientation ability score.

### Driving behaviour measures

As part of the Driving Habits Questionnaire (DHQ) [23], participants were asked “What is your annual mileage in a typical year?”. This comprised a driving frequency measure. Participants were also asked how often they drive within 6 geographical divisions, from within their immediate neighbourhood (lowest), to outside their region (highest). For each item, scores were rated from one (a few times in the year) to four (every day). Scores were totalled across all six items, and this measure comprised driving space.

### Analysis and statistics

Firstly, we estimated the prevalence of GPS usage within our older adult sample and assessed the association of binary GPS usage (use/no use) with demographic and driving variables using t-tests and chi-squared tests. We then assessed how frequency and situational usage of GPS related to demographic differences using linear regression (frequency) and multinomial logistic regression (situational usage). A post-hoc analysis was then conducted to assess how non-GPS in-vehicle technology usage relates to demographic variables to enable comparisons with GPS usage. ANCOVA was used to assess how situational usage of GPS related with cognitive functioning, and hierarchical regressions were conducted to assess the relationship between GPS frequency and cognitive functioning. MANCOVA was used to assess how situational usage affected driving mobility (driving frequency and space). In assessing the relationship between GPS use and cognitive variables, age and gender (0 = male, 1 = female) were used as covariates for analysis due to their previously established effects on spatial orientation and episodic memory. Age was also used as a covariate for analysis between GPS usage and driving behaviour as older age is associated with a reduced driving frequency and space. To establish whether GPS use can offset wayfinding impairments to improve driving mobility, we performed a median split on the allocentric orientation measure to define high and low navigational ability groups relative to the sample. Participants who scored higher than the median score (3.36) were in the low navigating ability group, and participants who scored below were in the high navigator group. It was then assessed whether worse navigators who use GPS have differences in driving frequency and driving space compared to better navigators. ANCOVAs were then employed to assess how driving mobility differed between both groups.

Outliers were assessed using boxplots, Q-Q plots, and histograms. Extreme outliers were removed for recognition memory (16), source memory (17), typical annual mileage (10), weekly trips (14), and weekly trip distance (11). A significance threshold of 0.05 was used to assess statistical significance. Tukey’s post-hoc comparisons were carried out to establish group differences in driving mobility and cognitive performance across GPS situational usage groups. Multinomial logistic regressions were used to assess how both demographic and cognitive variables predicted GPS behaviour. The reference group selected for each regression was individuals who do not use GPS, as this provided the greatest theoretical contrast to GPS usage situations. For MANCOVA analysis, checking normality of outcome variables was conducted using visual inspection of histograms and normality of residuals was conducted by QQ-Plots. For regression analysis, appropriate diagnostic tests and visual inspections were conducted to assess linearity and homoscedasticity, normality of residuals, independence of residuals, and multicollinearity. All analysis was carried out in R (version 4.3.1) using multcomp, nnet, olsrr, car, stats, and psych packages.

## Results

### The demographic patterns of GPS usage amongst older adult drivers

**Fig 1:**
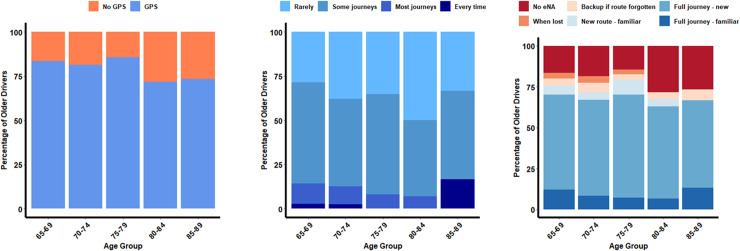
Frequency plots for GPS usage and frequency across older driver age groups.

Within our cohort, 82.35% of older drivers reported using GPS. Of the individuals who use Sat Nav, 53.63% reported using GPS on some journeys, 33.87% reported rarely using GPS, 10.08% reported using GPS on most journeys, and 2.42% reported using GPS every time they drive. The majority of drivers used GPS for the entire journey to a new destination (71.64%); followed by the entire journey along a familiar route (11.94%); on new routes to familiar destinations (6.65%); for backup in case of forgetting a route (5.70%); and then when lost on a new route (4.07%) (see Fig 1).

Individuals who use GPS reported a higher number of driving days per week (*M* = 4.27, SD = 1.59) compared to those who do not use GPS (*M* = 3.91, SD = 1.70), *p* =.02, d = 0.22 (small effect); as well as a greater number of trips per week (*M* = 2.01, SD = 1.86 compared to *M* = 1.61, SD = 1.22), *p* =.008, d = 0.23 (small effect); a higher typical annual mileage (*M* = 6923.47, SD = 3491.63 compared to *M* = 5475.45, SD = 3843.82), *p* <.001, d = 0.41 (medium effect); and had a greater driving space (*M* = 9.85, SD = 2.79 compared to *M* = 9.06, SD = 3.31), *p* =.006, d = 0.27 (small effect) than individuals who do not use GPS. Furthermore, individuals who use GPS used more of other in-vehicle technologies (*M* = 0.74, SD = 0.68) than individuals who do not use GPS (*M* = 0.24, SD = 0.43), *p* <. 001, d = 0.77 (strong effect) (see Table 1).

**Table 1:** Participant demographic and driving characteristics.

Variable	GPS usage			
	No GPS	GPS	*p*-value	Effect size *(d)*
Participants	158	737		
Age (years)	71.51 (5.25)	70.94 (4.90)	0.21	0.11
Education (years)	14.91 (2.63)	14.80 (2.77)	0.64	0.04
Driving experience (years)	48.62 (7.85)	49.35 (7.34)	0.29	0.10
Subjective driving ability	3.72 (0.66)	3.79 (0.64)	0.17	0.12
Weekly driving (days)	3.91 (1.70)	4.27 (1.59)	0.02	0.22
Typical mileage	5475.45 (3843.82)	6923.47 (3491.63)	<.001	0.41
Weekly trips	1.61 (1.22)	2.01 (1.86)	<.01	0.23
Driving space	9.06 (3.31)	9.85 (2.79)	<.01	0.27
Maximum weekly trip distance (miles)	9.22 (10.22)	9.59 (12.08)	0.77	0.03
Other in-vehicle technology usage	0.24 (0.43)	0.74 (0.68)	<.001	0.77

Welch’s two sample t test conducted for group differences. Cohen’s D was used to assess effect sizes.

A multiple regression was conducted to assess how age, gender, and education were associated with GPS frequency. Only being male gender was associated with increased GPS frequency (β = -0.24, *p* =.03, CI[-0.45, -0.02]) (see Table 2).

**Table 2:** Multiple linear regression analysis comparison between frequency of GPS usage and other IVT usage.

Variable	Age	Gender	Education	Model R^2^
GPS frequency	-0.01	-0.24*	0.02	0.02
Other IVT usage	-0.01**	-0.29***	-0.01	0.04

*p <.05, **p <.01, ***p <.001.

† IVT = In-vehicle technology.

†† Displaying unstandardised beta coefficients.

A multinomial logistic regression was conducted to assess how age, gender, and education predicted GPS situational usage. Being of early old age (expβ = 0.93, *p* <.01, CI[-0.13, -0.02]) and being of male gender (expβ = 0.25, *p* <.001, CI[-1.93, -0.81]) were associated with increased usage of GPS for the entire journey when driving to a familiar environment.

### The demographic patterns of wider in-vehicle technology usage amongst older drivers

A linear regression was then conducted to assess how non-GPS in-vehicle technology usage is associated with age, gender, and education. Being of early old age (β = -0.01, *p* = 0.001, CI[-0.02, -0.01]) and being of male gender (β = -0.29, *p <.*001, CI[-0.38, -0.20]) were associated with greater usage of other in-vehicle technology (see Table 2).

### How is GPS usage associated with driving behaviour?

A MANCOVA design was employed to assess how GPS situational usage influences driving frequency and driving space after controlling for age. There was a statistically significant difference between GPS situational usage groups on the combined outcome variables of driving space and driving frequency, *F*(5, 874) = 4.786, *p* < 0.001, η_p_^2^ (partial eta squared) =.05. Tukey’s post-hoc pairwise comparisons revealed that individuals who do not use GPS have a lower annual typical mileage (*M* = 5475.45, SD = 3843.82) than those who use their GPS when travelling new routes to a familiar destination (*M* = 7275.51, SD = 3830.55); for the entire journey to a new destination (*M* = 6685.28, SD = 3418.51), and for the entire journey to familiar destinations (*M* = 8521.60, SD = 3613.28). Individuals who used GPS for the entire journey to familiar destinations also had a higher typical mileage than those who used GPS for the entire journey when driving to new destinations and those who use their GPS when they are lost (*M* = 5933.33, SD = 3400.64). Tukey’s post-hoc comparisons also revealed that individuals who use GPS for the entire journey to familiar destinations also had a greater overall driving space (*M* = 10.63, SD = 2.47) than those who do not use GPS (*M* = 9.06, SD = 3.31) and those who use GPS only when lost (*M* = 8.90, SD = 2.81).

### How is GPS frequency associated with driving mobility?

A hierarchical regression design was then employed to establish whether GPS frequency is associated with driving mobility after controlling for age effects. More frequent GPS usage was associated with a greater typical annual mileage (β = 555.40, *p* = 0.001, CI [221.43, 889.32]) and greater driving space (β = 0.38, *p* = 0.008, CI [0.10, 0.66]).

### How is GPS frequency associated with cognitive performance?

A hierarchical regression design was employed to establish whether GPS frequency is associated with objective cognitive performance after controlling for age and gender effects. No objective cognitive functions were associated with increased GPS frequency.

A hierarchical regression model was employed to establish whether GPS frequency is associated with subjective navigation ability after controlling for age and gender effects. A worse subjective orientation ability was associated with greater GPS frequency when driving (β = -1.62, *p* = 0.003, CI [-2.70, -0.55]) (see Table 3).

**Table 3:** The association between cognitive ability and GPS usage.

	GPS usage categories						
Cognitive variable:	No GPS (1)	When lost (2)	Backup if route forgotten (3)	New route – familiar (4)	Full journey – new (5)	Full journey – familiar (6)	Sig. group differences
Allocentric orientation	4.09 (1.87)	3.56 (1.43)	3.72 (1.51)	4.15 (1.87)	3.64 (1.84)	3.25 (1.47)	–
Egocentric orientation	64.64 (34.40)	55.96 (35.48)	60.43 (29.57)	55.50 (26.56)	59.06 (35.26)	42.06 (28.28)	–
Recognition Memory	94.91 (6.13)	92.88 (8.18)	95.87 (4.11)	96.88 (3.51)	95.17 (5.62)	95.22 (5.80)	–
Source Memory	91.39 (9.94)	88.01 (13.23)	83.68 (14.91)	90.04 (11.71)	89.09 (12.38)	90.29 (12.15)	£^, $^
Subjective orientation ability	94.40 (10.19)	94.72 (12.00)	95.24 (11.43)	95.45 (11.28)	89.89 (11.69)	91.88 (11.38)	^%,?, €^
Landmark-based navigation usage	3.14 (0.54)	3.19 (0.52)	3.05 (0.54)	3.24 (0.54)	3.04 (0.53)	3.05 (0.49)	^!^

For Landmark-based navigation usage, all groups using GPS (2-6) were compared with the reference group of no GPS (1).

£ = (1) vs (3), *p* = 0.01; ^$^= (6) vs (3), *p* = 0.05; ^%^ = (4) vs (5), *p* < 0.01;^?^ = (1) vs (5), *p* <.001; ^€^ = (3) vs (5), *p* <.05;^!^ = (1) vs (5), *p* <.05.

### Is GPS situational usage associated with cognitive performance?

**Fig 2:**
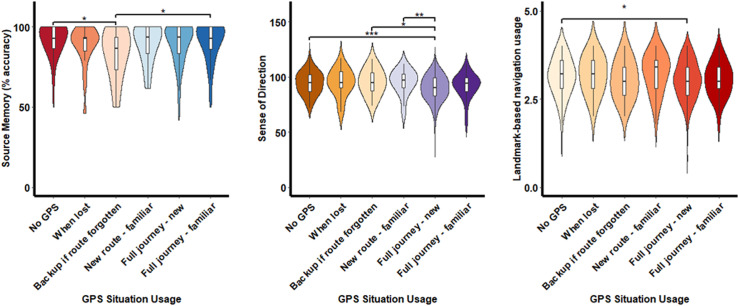
The association between GPS usage and cognitive ability.

An ANCOVA design with age and gender as covariates was carried out to establish how GPS situational usage is associated with objective cognitive performance (see Table 3). The only cognitive function with significant associations with GPS situational usage was source memory (*F*(5, 645) = 2.327, p = 0.04, η_p_^2^ = 0.018). Post hoc comparisons with a Tukey correction revealed significantly worse source memory performance in individuals who use GPS as backup in case they forget a route (*M* = 83.67, SD = 14.91) compared to individuals who do not use GPS (*M* = 91.39, SD = 9.94) and those who use GPS for everyday journeys to familiar destinations (*M* = 90.29, SD = 12.15) (see Fig 2).

An ANCOVA design with age and gender as covariates also revealed that subjective orientation ability is associated with GPS situational usage (*F*(5, 818) = 6.792, p < 0.001, η_p_^2^ = 0.04). Post hoc comparisons with a Tukey correction revealed that individuals who use GPS for the entire journey to new destinations have a subjectively worse sense of direction (*M* = 89.89, SD = 11.69) than individuals who use GPS when following a new route to a typical destination (*M* = 95.45, SD = 11.28), those who use GPS as a backup in case they forget a planned route (*M* = 95.24, SD = 11.43), and those who do not use GPS (*M* = 94.40, SD = 10.19) (see Fig 2).

### Are GPS frequency and situational usage associated with subjective spatial strategy?

A linear regression controlling for age and gender was conducted to establish whether GPS situational usage is associated with increased landmark-based navigation strategies. Only individuals who use GPS for everyday journeys to new destinations had a significant association with reduced landmark-based navigation strategies compared to individuals who do not use GPS (β = -0.10, *p* <.05, CI[-0.20, -0.00]) (see Fig 2).

A linear regression controlling for age and gender revealed that there was no association between GPS frequency and landmark-based navigation strategies.

### Can GPS usage ameliorate cognitive changes to improve driving mobility?

After defining high and low wayfinding groups, it was found that within the low wayfinding group, 161 older drivers used GPS compared to 44 who did not. An ANCOVA design revealed that among individuals with wayfinding impairments, those who use GPS have greater driving frequency than individuals who do not use GPS, *F*(1, 200) = 5.562, *p* = 0.02, η_p_^2^ =.03. Tukey’s post-hoc tests revealed that individuals who use GPS have a greater typical mileage (*M* = 6387.51, SD = 3264.98) than individuals who do not use GPS (*M* = 4994.36, SD = 3510.35).

**Fig 3:**
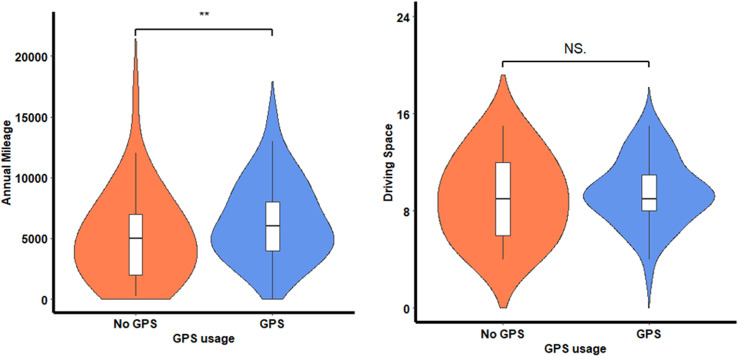
Driving mobility of older drivers with poor wayfinding ability split between GPS usage groups.

ANCOVA revealed no significant differences between the same groups for driving space (see Fig 3).

## Discussion

Overall, we found that a very high percentage (> 80%) of older people use GPS in car driving, and that drivers who use GPS more frequently have greater driving mobility. In particular, they drive greater distances and have a greater overall driving space even when reporting a reduced subjective sense of direction. Importantly, we demonstrate that GPS use facilitates driving mobility in individuals who show reduced objective wayfinding ability. We also expand on previous findings showing that the situations in which drivers rely on GPS are indicative of both subjective and objective cognitive differences.

In more detail, within our large community-dwelling sample of older adult drivers, we found that most older adults are users of GPS technology when driving (82.49%). These patterns complement previous findings within other older adult populations, where it has been found that GPS technology is commonly adopted amongst older drivers and have high acceptability rates [24]. We also found that older drivers typically use GPS for some driving journeys, with the least using it every time they drive, and that its situational usage was most often for the entire journey to a new destination. This is consistent with findings in a smaller sample of younger adults, where GPS was predominantly used for long and unique trips, and for approximately a quarter of trips [25].

Interestingly, a large US-based study [24] found that most older drivers used GPS always when driving, which may indicate there may be cultural differences influencing when older adults use GPS. One potential factor for this discrepancy may be driving distance, as within the US older drivers may be more likely to travel longer distances to reach their destination than in the UK, and therefore be more likely to use GPS for longer trips. Such potential country/culture specific differences need to be further explored in the future.

Our findings also contrast previous findings that older drivers are more likely to use GPS than younger drivers [15]. However, in the aforementioned study, older drivers were compared with younger driver populations and therefore variances across the older age spectrum were not tested for specifically. Furthermore, GPS usage has since become considerably more pervasive and is commonly pre-installed in modern vehicles, which has increased access to this technology across age groups. One potential reason why GPS usage is less prominent throughout the later older age spectrum may be due to difficulty in using the devices, as older populations may find difficulties in setting up and using GPS devices [12]. Early old age males were more likely to show greater dependence on the devices during driving, as they were significantly more likely to use them when navigating during the whole journey to familiar destinations. This relationship may be influenced by propensity to use in-vehicle technology in general, as we found that early old age males were also more likely to use other in-vehicle technologies, replicating previous survey findings [24]. Therefore, having a greater technological literacy may increase ease of use for adopting GPS technologies. Previous research indicates that older adults may struggle specifically with entering in destinations into GPS devices, but with live instruction can benefit substantially with improved driving performance when using GPS technology [9]. Older drivers may therefore benefit from instruction from occupational therapists and/or improved assistance from GPS developers to ensure that older drivers understand how to use navigation assistance systems to enable for greater driving mobility and improve driving performance.

Importantly, our study establishes for the first time that GPS technologies can offset age-related cognitive changes to facilitate greater driving mobility, as we found that older adults with poor navigation performance who use GPS have a greater annual mileage than those who do not use GPS. This supports the proposal that in-vehicular technologies can potentially compensate for age-related cognitive impairments in enabling driving mobility and independence in older drivers [6,8,26]. As worse wayfinding performance is associated with reductions in driving frequency and space [20,21], GPS technologies can aid in route guidance and reduce the cognitive load in navigating effectively. As older adults typically reduce their driving frequency and visits to unfamiliar locations in adapting to age-related changes, such as navigation concerns, the use of GPS technology devices should be recommended as a tool to prevent wayfinding deficiencies being an obstacle for mobility and independence. Furthermore, recent findings show that GPS devices can improve driving performance by reducing errors that may be caused by wayfinding burden within older adults [9,11,27]. Therefore, the implementation of GPS devices for individuals with wayfinding difficulties may also improve road safety within older adults - who are at greatest risk for fatal road traffic collisions [28]. By improving driver safety and driving mobility, the implementation of GPS technology may also potentially reduce driver cessation rates in older age.

When assessing the relationship between GPS usage and cognition, we found that increased GPS frequency and situational usage indicating greater GPS dependency (i.e., using GPS for the entire journey to new destinations) was associated with a reduced self-reported sense of direction within healthy older adults, but not objective spatial navigation impairments. Our results indicate that usage of GPS when driving may be determined by confidence in wayfinding successfully, as opposed to wayfinding ability. As GPS technology became more common in the 2000s [29], it is possible that older adults may have pre-formed their spatial cognitive abilities prior to commonplace GPS usage, and therefore current measures of spatial orientation performance may be representative of lifetime navigation skill, determined less by current GPS reliance. Furthermore, as early old age male drivers reported greater GPS use, and typically perform better in navigation than older age females [30], it is possible that this explains the lack of group differences in spatial navigation performance with GPS usage. Previous experimental studies, conducted amongst younger populations, have found that driving whilst using GPS is associated with impairments to route learning [31,32]. Therefore, whilst greater GPS usage may not impair objective cognitive facets overall, they may impair learning of spatial environments to which they are applied.

On a theoretical level, whilst several theories have postulated as to why GPS technology reduces survey knowledge acquired from the environment, including reduced cognitive load, divided attention, and reduced time to process environmental features due to increased velocity [32,33], the present study provides a potential explanation that survey knowledge is reduced due to spatial learning strategy, as we show that drivers who use GPS for everyday journeys to new locations were less likely to use landmark-dependent navigation strategies. Landmark-dependent strategies involve recognising and storing landmarks as reference points and are positively associated with better survey knowledge and cognitive map formation [34,35]. As GPS provide route guidance instructions in the form of turn-by-turn instructions, akin to sequence-based navigation strategies, it is possible that drivers who rely upon GPS technology for the entire journey to new destinations demonstrate reduced place-based spatial learning strategies as they are typically navigating to new destinations via response learning. Interestingly, a recent study found that providing landmark-based navigation assistance cues when navigating an unfamiliar environment can improve spatial knowledge acquisition of the environment [36]. Designers of navigation assistance systems may therefore look to integrate landmark-based cues to increase engagement with environmental surroundings.

Despite the exciting findings our study has some limitations. Firstly, drivers self-reported their usage of GPS systems as well as their driving mobility data. It is therefore possible that individuals may not be accurately estimating their driving frequency and space and may show individual differences in interpreting routes they use GPS for (e.g., there may be discrepancies as to how individuals perceive a familiar destination). However, virtually all previous studies have used self-reported usage measures, still future studies might use more automated data collection via GPS devices. Secondly, the present study did not account for other factors which may influence navigation behaviour, such as the presence of a co-pilot or passenger who may assist or hinder in navigational instructions, as well as the contributions of other in-vehicle technologies individually. Lastly, we did not investigate for potential differences in which types of navigation systems drivers use – such as app-based smartphone systems, specialised GPS devices, or built-in in-vehicle navigation devices, which may influence the frequency of GPS technology usage. Future studies should look at using driving diaries or in-vehicle cameras to complement naturalistic driving measurements to provide more granular information as to when older adults are attending to the devices during journeys and objective data regarding driving mobility. Such studies would provide greater objective clarity as to how GPS is used with specific driving situations, including how often older adults take new routes to familiar destinations. Furthermore, given that the present study demonstrates that GPS technology can facilitate driving in older adults with worse cognitive abilities, future studies should take an intervention approach in administering and training older adults how to use GPS devices to examine whether they improve driving mobility as well as performance.

In conclusion, this study provides valuable insights into understanding how the older adult population uses GPS technology, demonstrates that drivers with poorer wayfinding abilities can effectively use GPS technology to improve their driving mobility, and that how GPS technologies are used for specific journeys can indicate for subjective and objective cognitive differences, including navigation strategies.

## Supporting information

S1 Appendix The Driving, Orientating, and Navigating questionnaire (DON). (DOCX)

S1 Table Summary statistics of cognitive test performance.
(DOCX)
