# Supplementary material for: GPS navigation assistance is associated with driving mobility in older drivers
Source: PLOS Digit Health. 2025 Apr 3;4(4):e0000768. doi: 10.1371/journal.pdig.0000768 (PMC11967921; doi:10.1371/journal.pdig.0000768)
Supplement: S1 Appendix — (DOCX) [file pdig.0000768.s001.docx]

**S1 Appendix**

**The Driving, Orientating, and Navigating questionnaire (DON)**

*Note.* When scoring the DON, Q4; Q7; Q8, Q10; Q15; Q18; Q19; Q20; Q21; Q23; Q24; Q28 are reverse scored before totalling.

For the present manuscript, Q11; Q20; Q21; Q26; Q30 were used to comprise the landmark spatial strategy usage.

| **Driving, Orientating, and Navigating questionnaire** | | | | | | |
| --- | --- | --- | --- | --- | --- | --- |
|  |  | **Never** | **Rarely** | **Sometimes** | **Often** | **Always** |
| **1** | Whilst on a familiar route, I know the general direction of my destination |  |  |  |  |  |
| **2** | If my regular route were blocked, e.g. because of road works, I could easily find an alternative route |  |  |  |  |  |
| **3** | Once I have learned a route, I don’t need to know exactly where I am, as long as I can reach my destination |  |  |  |  |  |
| **4** | When driving along a route I know well, I have made a wrong turn because I mistook my location |  |  |  |  |  |
| **5** | Even if I were unsure of my precise location on a new route, I would still know the general direction of my destination |  |  |  |  |  |
| **6** | When learning a new route, I orient myself according to street layouts |  |  |  |  |  |
| **7** | When driving to a new destination, I have found myself in the wrong place because I missed a turn |  |  |  |  |  |
| **8** | If I were to break down on a new route, I would be unsure whether I was closer to home, or closer to my destination |  |  |  |  |  |
| **9** | When imagining a familiar route, I think in terms of compass directions (N, S, E, W) |  |  |  |  |  |
| **10** | I have got lost on an unfamiliar route, and have required assistance to reach my destination |  |  |  |  |  |
| **11** | Along unfamiliar routes, I pay attention to my surroundings, e.g. buildings, shops, trees |  |  |  |  |  |
| **12** | When learning a new route, I try to keep track of my current location throughout the journey |  |  |  |  |  |
|  |  | **Never** | **Rarely** | **Sometimes** | **Often** | **Always** |
| **13** | I navigate new routes in terms of compass directions (N, S, E, W) |  |  |  |  |  |
| **14** | I give detailed instructions when people ask me for directions |  |  |  |  |  |
| **15** | I have got lost on a familiar route, and have required assistance to reach my destination |  |  |  |  |  |
| **16** | Even if I were unsure of my precise location on a new route, I would still know the general direction of my starting point |  |  |  |  |  |
| **17** | On familiar routes, I get my bearings from street layouts, e.g. corners, signs, junctions |  |  |  |  |  |
| **18** | When driving along a route I know well, I have missed a turn because my mind was elsewhere |  |  |  |  |  |
| **19** | If I were to breakdown on a familiar route, I would have difficulty providing directions to the recovery vehicle to get me home |  |  |  |  |  |
| **20** | Along familiar routes, I am unaware of changes in my surroundings, e.g. new buildings, autumn leaves |  |  |  |  |  |
| **21** | When driving along a regular route, well-known landmarks have felt unfamiliar |  |  |  |  |  |
| **22** | If I needed to stop somewhere along a new route, e.g. to check my location, I could find my current location on a map |  |  |  |  |  |
| **23** | If someone asks me for directions, I point them the right way, but cannot give precise details |  |  |  |  |  |
| **24** | When driving along a regular route, street layouts have felt different, even though I know they haven’t changed |  |  |  |  |  |
| **25** | At any given point along a familiar route, I know roughly how far I am away from my starting point |  |  |  |  |  |
|  |  | **Never** | **Rarely** | **Sometimes** | **Often** | **Always** |
| **26** | On familiar routes, I get my bearings from distinct landmarks (e.g. churches, pubs, shops) |  |  |  |  |  |
| **27** | If I were to stop somewhere along a familiar route, I could locate myself on a map |  |  |  |  |  |
| **28** | When driving to a new destination, I have found myself in the wrong place because I turned off too soon |  |  |  |  |  |
| **29** | Whilst on a familiar route, I know the general direction of my starting point |  |  |  |  |  |
| **30** | When learning a new route, I orient myself according to distinct landmarks |  |  |  |  |  |
| **31** | I can remember a new route after I have travelled it only once |  |  |  |  |  |
