# Supplementary material for: GPS navigation assistance is associated with driving mobility in older drivers
Source: PLOS Digit Health. 2025 Apr 3;4(4):e0000768. doi: 10.1371/journal.pdig.0000768 (PMC11967921; doi:10.1371/journal.pdig.0000768)
Supplement: S1 Table — (DOCX) [file pdig.0000768.s002.docx]

| **S1 Table. Summary statistics of cognitive test performance** | | | |
| --- | --- | --- | --- |
| **Cognitive test** | **Range** | **Median** | **Mean** |
| Allocentric Orientation | 0 - 9.54 | 3.36 | 3.71 |
| Egocentric Orientation | 7.97 – 155.94 | 49.83 | 57.51 |
| Recognition Memory | 70.00% – 100% | 96.67% | 95.18% |
| Source Memory | 41.67% - 100% | 92.86% | 89.36% |
| DON | 36 - 121 | 93.00 | 91.61 |
| Note. Higher value of Allocentric orientation and Egocentric orientation indicate greater error, higher value of Source Memory, Recognition Memory, and DON indicate for better performance. | | | |
